# Receptor guanylyl cyclase Gyc76C is required for invagination, collective migration and lumen shape in the *Drosophila* embryonic salivary gland

**DOI:** 10.1242/bio.20134887

**Published:** 2013-05-29

**Authors:** Unisha Patel, Monn Monn Myat

**Affiliations:** Department of Cell and Developmental Biology, Weill Cornell Medical College, 1300 York Avenue, New York, NY 10065, USA

**Keywords:** *Drosophila*, Guanylyl cyclase, Integrin, Migration, Morphogenesis, Salivary gland

## Abstract

The *Drosophila* embryonic salivary gland is formed by the invagination and collective migration of cells. Here, we report on a novel developmental role for receptor-type guanylyl cyclase at 76C, Gyc76C, in morphogenesis of the salivary gland. We demonstrate that Gyc76C and downstream cGMP-dependent protein kinase 1 (DG1) function in the gland and surrounding mesoderm to control invagination, collective migration and lumen shape. Loss of *gyc76C* resulted in glands that failed to invaginate, complete posterior migration and had branched lumens. Salivary gland migration defects of *gyc76C* mutant embryos were rescued by expression of wild-type *gyc76C* specifically in the gland or surrounding mesoderm, whereas invagination defects were rescued primarily by expression in the gland. In migrating salivary glands of *gyc76C* mutant embryos, integrin subunits localized normally to gland–mesoderm contact sites but talin localization in the surrounding circular visceral mesoderm and fat body was altered. The extracellular matrix protein, laminin, also failed to accumulate around the migrating salivary gland of *gyc76C* mutant embryos, and *gyc76C* and *laminin* genetically interacted in gland migration. Our studies suggest that *gyc76C* controls salivary gland invagination, collective migration and lumen shape, in part by regulating the localization of talin and the laminin matrix.

## Introduction

Salivary glands of the *Drosophila* embryo consist of a pair of elongated epithelial tubes, formed by the invagination of primordial cells from the ventral surface of the embryo. Salivary gland cells invaginate in a coordinated and sequential manner through apical constriction and basal nuclear migration (Maruyama and Andrew, 2012; Pirraglia and Myat, 2010). Upon completion of invagination, the gland migrates dorsally to contact the overlying circular visceral mesoderm (CVM), at which point the gland turns and migrates posteriorly through continued contact with the CVM and with the underlying somatic mesoderm (SM) and fat body (FB) (Bradley et al., 2003; Vining et al., 2005). Salivary gland cells migrate in a collective manner while maintaining cell–cell contacts, apical–basal polarity, and in the absence of cell proliferation and cell death. Distal cells of the salivary gland are the first cells to contact the CVM and migrate by extending basal membrane protrusions and elongating in the direction of migration (Bradley et al., 2003; Pirraglia and Myat, 2010; Pirraglia et al., 2013). This is distinct from the manner in which the proximal salivary gland cells migrate; proximal cells change shape from columnar to cuboidal and rearrange as they migrate dorsally and turn posteriorly (Xu et al., 2011; Xu et al., 2008).

Posterior turning and migration of the salivary gland is dependent on integrin mediated contact between the gland and surrounding tissues. The αPS1 integrin subunit, encoded by *multiple edematous wings* (*mew*) is expressed in the salivary gland , whereas the αPS2 subunit, encoded by *inflated*, (*if*), is expressed in the surrounding CVM, SM and FB (Bradley et al., 2003). During salivary gland migration, the αPS2 and βPS integrin subunits accumulate at sites of contact between the gland and surrounding tissues (Jattani et al., 2009). In embryos mutant for *mew*, *if* or *myospheroid*, encoding the βPS subunit, salivary glands fail to contact the surrounding tissues and fail to migrate (Bradley et al., 2003; Vining et al., 2005). Integrin mediated adhesion between the salivary gland and surrounding tissues leads to recruitment of the small GTPase Rac1 to the basal membrane of gland cells at gland–mesoderm contact sites and its possible activation (Pirraglia et al., 2013). In the distal salivary gland cells, Rac1 and Rac2 GTPases downregulate the cell–cell adhesion protein, E-cadherin, and promote cell elongation and basal membrane protrusion in the direction of migration (Pirraglia et al., 2006; Pirraglia et al., 2013). Salivary gland migration also requires a laminin matrix (Ismat et al., 2013; Urbano et al., 2009). The *Drosophila* genome encodes two α chains (α1,2 and α3,5), one β chain and one γ chain that assemble into two laminin trimers, lamininA (α3,5; β1;γ1) and lamininW (α1,2;β1;γ1). Laminin chains are expressed in the visceral mesoderm and somatic mesoderm surrounding the salivary gland and are required for the migration of a number of tissues other than the gland (Martin et al., 1999; Montell and Goodman, 1989; Urbano et al., 2011; Urbano et al., 2009; Wolfstetter and Holz, 2012).

The salivary gland lumen is formed as gland cells invaginate to form a tubular organ; however, lumen size changes concomitant with gland migration. Lumen width decreases specifically in the proximal region of the gland and lumen length increases as the gland migrates (Pirraglia et al., 2010). Numerous mechanisms exist for regulating salivary gland lumen size and shape. These include Rho1-dependent control of the actin cytoskeleton (Xu et al., 2011), p21 activated kinase 1 (Pak1)-dependent control of E-cadherin endocytosis (Pirraglia et al., 2010), Rac1-dependent control of cell rearrangement, cell elongation and basal membrane protrusion (Pirraglia et al., 2013), and Hairy-dependent control of apical membrane growth and delivery (Myat and Andrew, 2002). Additionally, recent studies of ADAMTS-A, a member of the ADAMTS family of secreted metalloproteases, suggest a role for the secreted apical extracellular matrix in control of salivary gland lumen shape (Ismat et al., 2013).

From a large-scale chemical mutagenesis screen designed to identify mutations affecting salivary gland and tracheal morphogenesis, we generated a novel allele of guanylyl cyclase at 76C, *gyc76C^2388^* (Myat et al., 2005; Patel et al., 2012). Guanylyl cyclases (GCs) catalyze the conversion of GTP to cGMP (guanosine 3′, 5′-cyclic monophosphate) in response to signals such as nitric oxide (NO), peptide ligands and changes in intracellular calcium. cGMP generated by soluble and receptor-type GCs regulate cellular events by activating cGMP-dependent protein kinases (cGKs or PKGs), ion channels or phosphodiesterases (Davies, 2006; Lucas et al., 2000; Morton, 2004). PKGs represent the major intracellular effectors of cGMP signaling (Davies, 2006; Lucas et al., 2000). In *Drosophila*, *pkg21D* (*dg1*) and *foraging* (*for, dg2*) encode the two cGMP-dependent kinases, DG1 and DG2, respectively. DG1 and DG2 modulate epithelial fluid transport by the Malpighian (renal) tubules (MacPherson et al., 2004), and mouse knock-outs of cGKII, the mammalian homolog of DG2, result in intestinal secretory defects (Pfeifer et al., 1996), suggesting that some physiological functions of cGKs may be conserved between *Drosophila* and mammals.

In *Drosophila* neurogenesis, *gyc76C* is required for axon pathfinding (Ayoob et al., 2004). We showed that in the *Drosophila* embryonic muscle, *gyc76C* is required for integrin receptor localization at sites of contact between the developing myotubes and tendon cells (Patel et al., 2012). Here, we report on a novel role for *gyc76C* in salivary gland invagination, migration and lumen shape, in part by regulating localization of the laminin matrix and talin.

## Results

### *gyc76C* is required for salivary gland invagination and migration

**Fig. 1.**
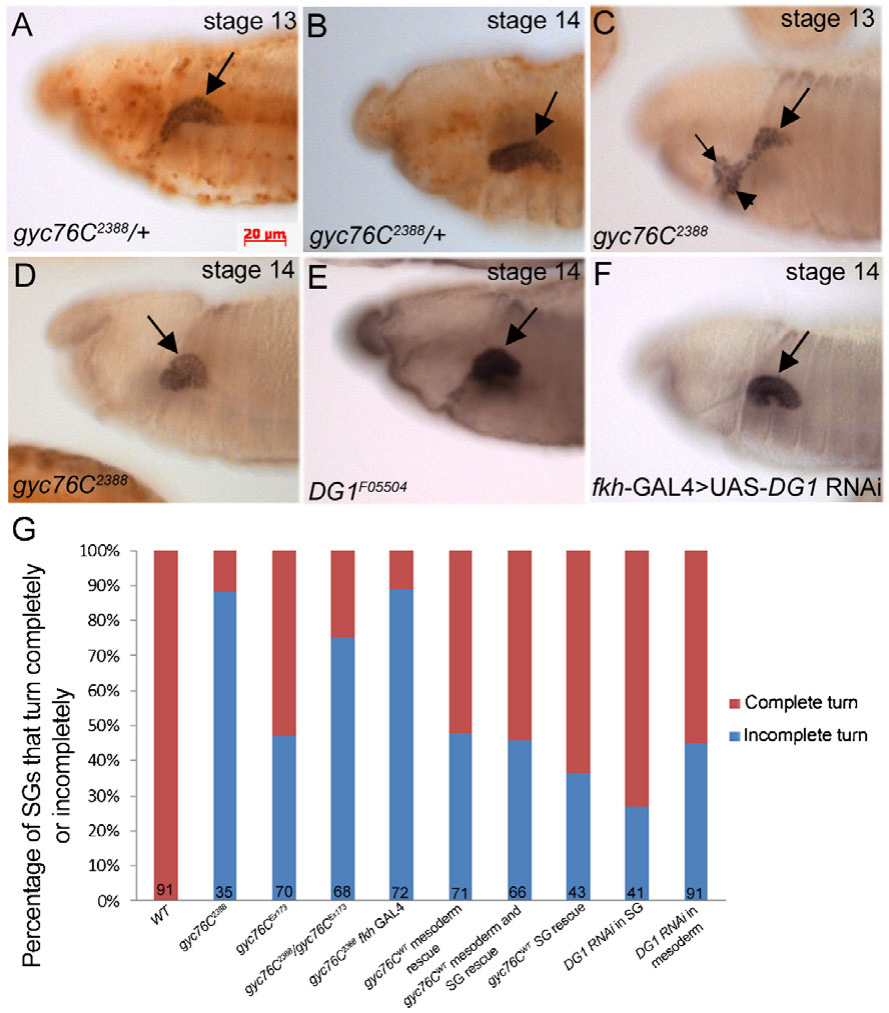
Salivary gland migration defects in *gyc76C* and *DG1* mutant embryos. In *gyc76C^2388^* heterozygous embryos (**A**,**B**), the salivary gland migrates posteriorly during stages 13 (A) and 14 (B). In *gyc76C^2388^* homozygous embryos (**C**,**D**) at stage 13 (C) and 14 (D), salivary glands do not complete their posterior migration (C,D, large arrows) with some proximal cells failing to invaginate (C, arrowhead) and the gland is branched (C, small arrow) or folded (D, arrow). In embryos homozygous for *DG1^F05504^* (**E**) or wild-type embryos expressing *DG1* RNAi specifically in the gland (**F**), salivary glands do not migrate completely (E,F, arrows). Graph depicting extent of salivary gland migration. Numbers indicate number of glands scored (**G**). All embryos shown were stained for dCREB whereas embryos in A–D were also stained for β-galactosidase (β-gal) to distinguish between heterozygous and homozygous embryos. SG: salivary gland. Scale bar: 20 µm.

To determine a role for *gyc76C* in salivary gland morphogenesis we analyzed gland invagination and migration in embryos mutant for a null allele of *gyc76C*, *gyc76C^2388^*, which is thought to lack the guanylyl cylcase domain (Patel et al., 2012). In *gyc76C^2388^* heterozygous embryos, all salivary gland cells invaginated to form a gland that turned posteriorly during stages 13 and 14 (Fig. 1A,B,G, Fig. 5G). By contrast, in *gyc76C^2388^* homozygous embryos, 16% of mutant glands did not invaginate completely with some cells remaining at the ventral surface (Fig. 1C, Fig. 5G). *gyc76C^2388^* homozygous embryos showed a severe salivary gland migration defect where 88% of mutant glands failed to turn completely and some glands appeared branched or folded (Fig. 1C,D,G). We observed similar defects in embryos homozygous for *gyc76C^Ex173^*, an allele in which about 8 kb of genomic DNA including a *gyc76C* exon are deleted by imprecise P-element excision (Ayoob et al., 2004) and embryos *trans*-heterozygous for *gyc76C^2388^* and *gyc76C^Ex173^*, though not to the same severity as in *gyc76C^2388^* homozygous embryos (Fig. 1G).

We previously showed by whole mount in situ hybridization that *gyc76C* RNA is enriched in the CVM and FB surrounding the migrating salivary gland and in the mature gland (Patel et al., 2012). To determine whether *gyc76C* is required in the salivary gland or in the CVM/FB for gland invagination and migration, we expressed wild-type *gyc76C*, *gyc76C^WT^* in either the gland or mesoderm, with *fkh*-GAL4 and *twi*-GAL4, respectively, or in both. Expression of *gyc76C^WT^* in just the salivary gland or mesoderm significantly rescued the gland migration defect of *gyc76C^2388^* homozygous embryos (Fig. 1G). Simultaneous expression of *gyc76C^WT^* in the salivary gland and mesoderm did not result in increased rescue of the migration defect compared to expression in either tissue alone (Fig. 1G). In contrast to the rescue of the migration defects, the salivary gland invagination defects of *gyc76C* mutant embryos were better rescued with expression of *gyc76C^WT^* in the gland than in the mesoderm (Fig. 5G).

To determine whether *pkg21D*, encoding cGMP-dependent protein kinase 1 (DG1) is required for salivary gland migration we analyzed embryos mutant for *DG1^f05504^* or embryos expressing *DG1* RNAi specifically in the gland or in the mesoderm with *fkh*-GAL4 and *twi*-GAL4, respectively. In *DG1^f05504^* mutant embryos, as in embryos expressing *DG1* RNAi, salivary gland migration was inhibited, although to a lesser extent than in *gyc76C^2388^* mutant embryos (Fig. 1E–G). We did not detect salivary gland defects in embryos mutant for DG2 (data not shown). From these data, we conclude that *gyc76C* is required in both the salivary gland and surrounding mesoderm for invagination and collective migration of the gland, and DG1 likely acts downstream of Gyc76C.

### Loss of *gyc76C* results in branching of the salivary gland lumen

**Fig. 2.**
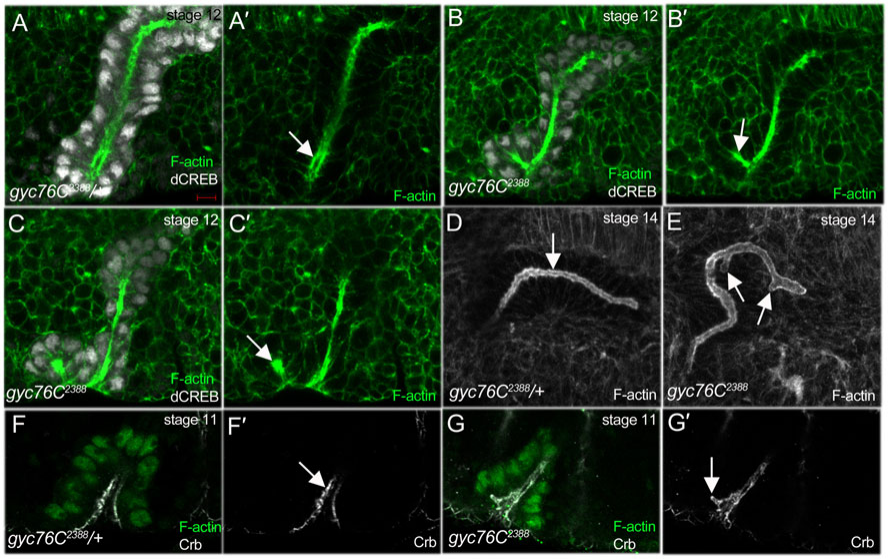
Salivary gland lumen shape defects in *gyc76C* mutant embryos. In *gyc76C^2388^* heterozygous embryos at stage 12 (**A**) and 14 (**D**), the salivary gland has a single central lumen (**A′**,D, arrows) whereas, in homozygous siblings at stage 12 (**B**,**C**), the gland lumen branches off the central lumen (**B′**, arrow) or forms through an ectopic invagination site (**C′**, arrow). In stage 14 *gyc76C^2388^* homozygous embryos (**E**), ectopic lumens form along the central lumen (E, arrows). In invaginating salivary glands of *gyc76C^2388^*heterozygous embryos (**F**), apical membranes of all cells are uniform and face the central lumen (**F′**, arrow) whereas, in glands of homozygous siblings (**G**), the apical membrane expands off the central lumen (**G′**, arrow). Embryos in A–C were stained for dCREB (white) to label salivary gland nuclei, phalloidin to label F-actin (green) and β-gal (not shown). Embryos in D,E were stained for F-actin with phalloidin and β-gal (not shown). Embryos in F,G were stained for Crb (white) to label the apical membrane dCREB (green) and β-gal (not shown). All images shown are one-µm thick optical sections, except for D,E which are projected images of seven one-µm thick optical sections. Scale bar: 5 µm.

Salivary gland invagination and migration defects in *gyc76C* mutant embryos were accompanied by defects in lumen shape. In *gyc76C^2388^* heterozygous embryos, the salivary gland lumen is a single continuous structure formed during the process of invagination (Fig. 2A,D,F). By contrast, in *gyc76C^2388^* homozygous embryos, the salivary gland lumen was branched with ectopic lumens along the length of the central lumen (Fig. 2B,E). To determine how the branching of the lumen originated, we analyzed the lumen of *gyc76C^2388^* mutant salivary glands during the invagination stage. We observed indentations of the apical membrane as *gyc76C^2388^* mutant salivary glands cells invaginated (Fig. 2G). We also observed an ectopic lumen arising from an ectopic invagination site anterior to the central lumen (Fig. 2C). The presence of the apical membrane protein, Crumbs (Crb) confirmed that apical–basal polarity was maintained in the branched lumens of *gyc76C^2388^* mutant salivary glands (Fig. 2G). These observations suggest that branching of the salivary gland lumen in *gyc76C* mutant embryos occurs through indentation of the apical membrane during invagination or through formation of an ectopic invagination site.

### Talin localization is altered in *gyc76C* mutant embryos

**Fig. 3.**
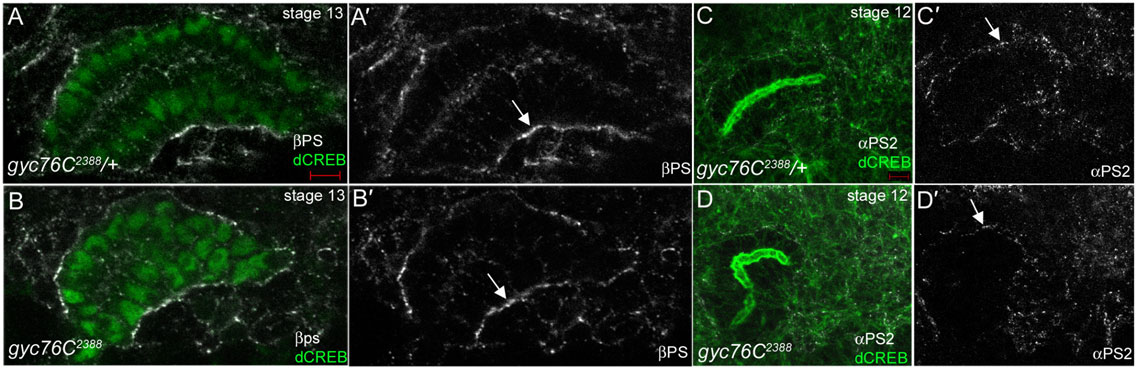
Loss of *gyc76C* does not affect βPS or αPS2 integrin localization during salivary gland migration. In stage 13 *gyc76C^2388^* heterozygous (**A**) and homozygous (**B**) embryos, βPS integrin is enriched at sites of contact between the gland and underlying FB (**A′**,**B′**, arrows). In *gyc76C^2388^* heterozygous (**C**) and homozygous (**D**) embryos, αPS2 is localized as puncta at gland–CVM contact sites (**C′**,**D′**, arrows). Embryos in A,B were stained for dCREB (green), βPS (white) and β-gal (not shown) whereas, embryos in C,D were stained for F-actin with phalloidin (green), αPS2 (white) and β-gal (not shown). Scale bar: 5 µm.

**Fig. 4.**
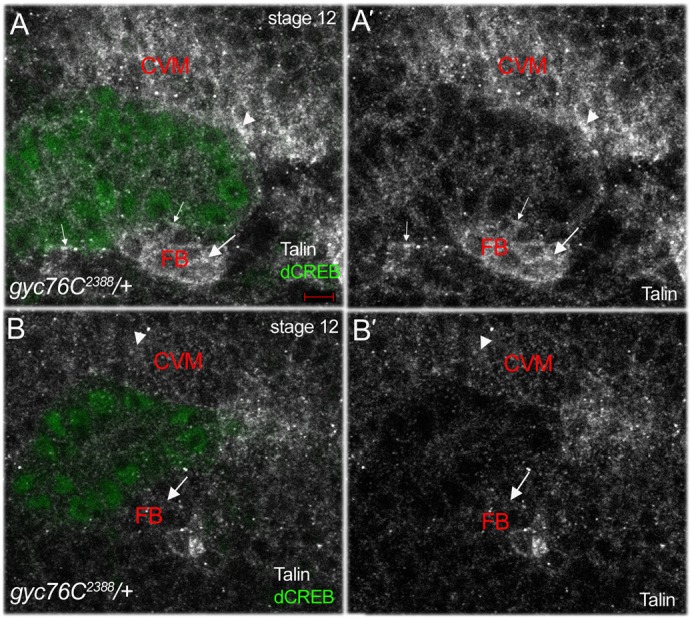
Talin is not enriched in the mesoderm of *gyc76C* mutant embryos. In *gyc76C^2388^* heterozygous embryos (**A**), talin is enriched in the FB underlying the migrating salivary gland (A,**A′**, large arrows), in the CVM overlying the gland (A,A′, arrowheads) and at gland–FB/SM contact sites (A,A′, small arrows). In *gyc76C^2388^* homozygous embryos (**B**) talin is not enriched in the FB (B,**B′**, arrows) and in the CVM (B,B′, arrowheads). Embryos were stained for Talin (white), dCREB (green) and β-gal (not shown). Scale bar: 5 µm.

During salivary gland migration, the αPS2βPS integrins accumulate at sites of contact between the gland and overlying CVM (gland–cVM) and the gland and underlying SM and FB (gland–SM/FB) (Jattani et al., 2009). Because *gyc76C* controls integrin receptor localization at MTJs during *Drosophila* myogenesis (Patel et al., 2012), we analyzed the localization of the βPS and αPS2 integrin subunits during salivary gland migration. In stage 13 *gyc76C^2388^* heterozygous and homozygous embryos, the βPS integrin subunit was enriched at the gland–FB contact sites (Fig. 3A,B). Similarly, the αPS2 integrin subunit was found as puncta at contact sites between the salivary gland and surrounding mesoderm in *gyc76C^2388^* homozygous embryos as in heterozygous embryos (Fig. 3C,D). However, we did detect changes in localization of Talin, a cytoplasmic linker protein that binds integrins and is essential for integrin activity (Brown et al., 2002). In *gyc76C^2388^* heterozygous embryos, Talin was enriched in the CVM overlying the migrating salivary gland, in the FB underlying the gland, and at gland–CVM and gland–FB contact sites (Fig. 4A,A′). By contrast, in *gyc76C^2388^* homozygous embryos, Talin was not enriched in the surrounding tissues and did not localize to gland–CVM or gland–FB contact sites (Fig. 4B,B′). Thus, although *gyc76C* was not required for integrin localization at gland–mesoderm contact sites, it is required for enrichment of Talin in the mesoderm surrounding the gland.

### Gyc76C is required for the accumulation of a laminin matrix

**Fig. 5.**
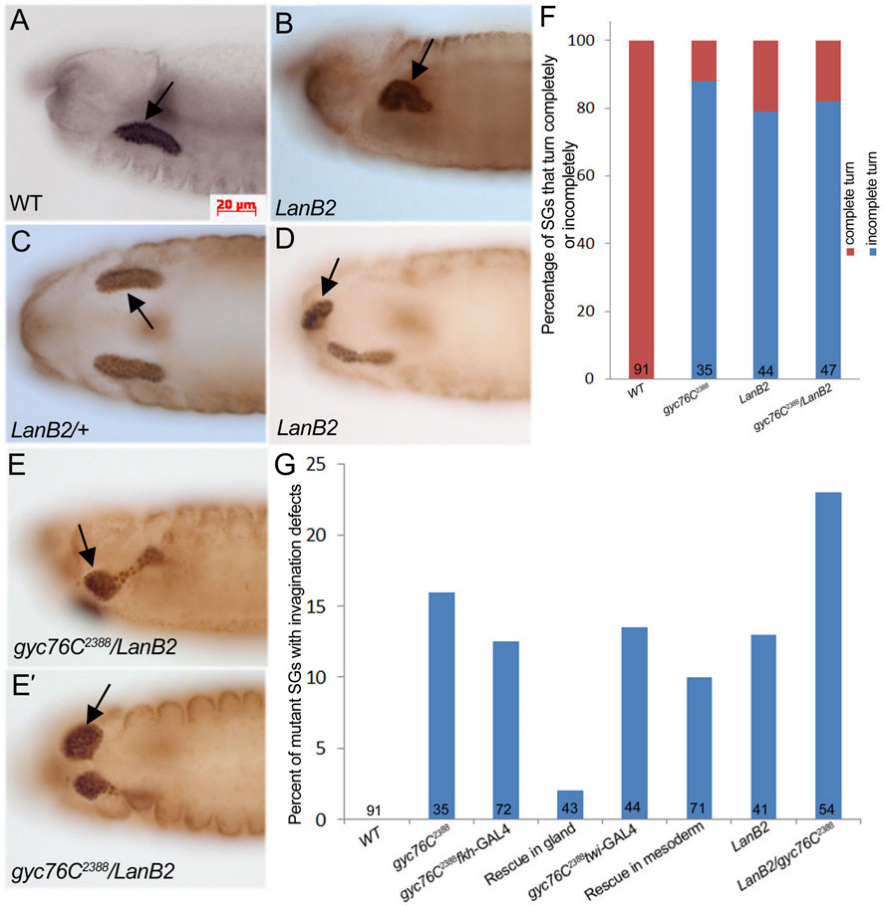
*gyc76C* genetically interacts with *LanB2* to control salivary gland invagination and migration. In wild-type embryos at stage 14 (**A**) the salivary gland turns completely (A, arrow), whereas in embryos mutant for *LanB2* (**B**), the gland does not complete its posterior turn (B, arrow). In *LanB2* heterozygous embryos at stage 15 (**C**), salivary gland migration is complete (C, arrow) whereas in homozygous siblings (**D**), gland cells do not invaginate completely from the embryo surface (D, arrow). In embryos *trans*-heterozygous for *gyc76C^2388^* and *lanB2* at stage 14 (**E**), salivary gland cells fail to invaginate from the embryo surface (E,**E′**, arrows). Graph depicting percentage of mutant salivary glands that turn completely or incompletely at stage 14. Numbers indicate number of glands scored (**F**). Graph depicting percentage of mutant salivary glands with invagination defects at stage 14. Numbers indicate number of glands scored (**G**). All embryos were stained for dCREB and embryos in B–E were also stained for β-gal. Scale bar: 20 µm.

In embryos mutant for *wing blister*, encoding the α1,2 laminin chain, or *lanB1* encoding the β chain, salivary glands do not migrate properly (Ismat et al., 2013; Urbano et al., 2009). We observed that in embryos mutant for *lanB2*, encoding the laminin γ chain, proximal gland cells did not complete their posterior turn, resulting in folded glands (Fig. 5B). Quantification of the salivary gland migration defect showed that 79% of *lanB2* mutant glands failed to complete their posterior migration (Fig. 5F). Similarly, 82% of glands of *gyc76C^2388^* and *lanB2 trans*-heterozygous embryos did not migrate completely, demonstrating a genetic interaction between *gyc76C* and *lanB2* (Fig. 5E,E′,F). *LanB2* mutant embryos also showed salivary gland invagination defects where proximal gland cells remained at the ventral surface of the embryo (Fig. 5D,G). Salivary glands of *LanB2* mutant embryos that failed to invaginate did not have branched lumens, unlike *gyc76C^2388^* mutant glands (data not shown). Simultaneous reduction of the gene dosage of both *gyc76C* and *lanB2* exacerbated the salivary gland invagination defect such that now 25% of mutant glands failed to invaginate compared to 16% in *gyc76C^2388^* and 13% in *lanB2* mutant glands (Fig. 5E,E′,G). Embryos mutant for *LanA^9–32^* encoding the α3,5 laminin chain showed no salivary gland invagination or migration defects (data not shown), suggesting that lamininW is the main laminin trimer involved in salivary gland migration.

**Fig. 6.**
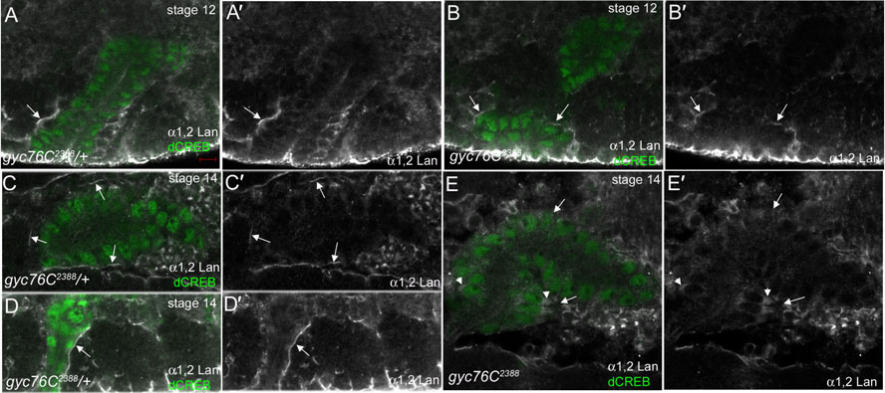
*gyc76C* mutant embryos fail to accumulate a laminin matrix around the migrating salivary gland. In migrating salivary glands of *gyc76C^2388^* heterozygous embryos at stage 12 (**A**) and stage 14 (**C**,**D**), the α1,2 laminin chain (α1,2Lan) is enriched around the proximal gland cells (A,**A′**,D,**D′**, arrows) and the medial gland cells that are in contact with surrounding tissues (C,**C′**, arrows). In *gyc76C^2388^*homozygous embryos at stage 12 (**B**), α1,2Lan localized around the proximal gland cells (B,**B′**, arrows). In *gyc76C^2388^*homozygous embryos at stage 14 (**E**), α1,2Lan did not localize to contact sites between the gland and surrounding tissues (E,**E′**, arrows) and instead localized to the basal cytoplasm of the proximal gland cells (E, E′, arrowheads). All embryos were stained for α1,2Lan (white), dCREB (green) and β-gal (not shown). Scale bar: 5 µm.

The genetic interaction observed between *gyc76C* and *lanB2* suggests that *gyc76C* controls salivary gland migration at least partly through regulation of the laminin matrix. To test this hypothesis, we stained for the α1,2 laminin chain (referred to here as α1,2Lan) in *gyc76C* mutant embryos. In *gyc76C^2388^* heterozygous embryos at stage 12, α1,2Lan was enriched at the basal membrane of migrating proximal gland cells and later at contact sites between the gland and surrounding tissues (Fig. 6A,C,D). In *gyc76C^2388^* homozygous embryos at stage 12, α1,2Lan localized to the basal membrane of the proximal gland cells (Fig. 6B). However, by stage 14, no α1,2Lan was detected around the salivary gland of *gyc76C^2388^* homozygous embryos although it was found in the basal cytoplasm of the mutant gland cells (Fig. 6E). These data suggest that *gyc76C* is required for the accumulation of a laminin matrix surrounding the migrating salivary gland.

## Discussion

In this study, we demonstrate a novel role for *gyc76C* in invagination, collective migration and lumen shape control of the *Drosophila* embryonic salivary gland. We show that *gyc76C* functions in both the gland and the surrounding mesoderm to regulate gland invagination and migration, in part by controlling talin and laminin localization. We previously reported on a role for *gyc76C* in localization of integrin receptors at myotendinous junctions (MTJ) during *Drosophila* myogenesis (Patel et al., 2012). Although we did not detect changes in αPS2 or βPS integrin localization at gland–mesoderm contact sites in *gyc76C* mutant embryos, this could be due to the maternal supply of *gyc76C*. In *gyc76C* mutant salivary glands, α1,2Lan did not accumulate in the matrix between the salivary gland and mesoderm and instead was found in the basal cytoplasm of the mutant gland cells. It is possible that *gyc76C* mutant salivary gland cells fail to secrete the laminin chains to assemble a laminin matrix. This is consistent with our previous report that in the myotubes of *gyc76C^2388^* mutant embryos, βPS integrin accumulates as puncta in the cytoplasm instead of being enriched at the MTJs (Patel et al., 2012). Thus, *gyc76C* may play a role in membrane trafficking events important for integrin-mediated adhesion in salivary gland migration and myogenesis.

Integrin function is required for the accumulation of a laminin-containing matrix during dorsal closure (Narasimha and Brown, 2004), in visceral mesoderm migration (Urbano et al., 2011) and in the developing gonad, (Tanentzapf et al., 2007). Additionally, the ECM induces conformational changes in integrins that regulate integrin activity through outside-in signaling (Giancotti and Ruoslahti, 1999; Pines et al., 2012; Qin et al., 2004). Because *gyc76C* is required in both the salivary gland and mesoderm for gland migration, the primary defect in *gyc76C* mutant embryos may be accumulation of a laminin matrix which affects integrin activation, or alternatively, talin-dependent integrin activation may be the primary defect which then affects accumulation of a laminin matrix. We currently cannot distinguish between these two possibilities.

We showed in this study that α1,2Lan was localized to the basal membrane of salivary gland cells, and glands failed to invaginate in *lanB2* mutant embryos, and embryos *trans*-heterozygous for *lanB2* and *gyc76C^2388^*. However, loss of integrin function does not disrupt salivary gland invagination (Bradley et al., 2003), suggesting that the laminin matrix regulates gland invagination in an integrin-independent manner. In addition to integrins, laminins bind Dystroglycan (DG), a widely-expressed ECM receptor (Yurchenco, 2011). DG is expressed in the embryonic gland (Shcherbata et al., 2007) and loss of DG in *Drosophila* follicle epithelial cells results in a reduced and misorganized laminin matrix (Deng et al., 2003). It will be of interest to test in future studies whether DG plays a role in salivary gland invagination and whether *gyc76C* regulates gland invagination through DG.

Numerous studies demonstrate a role for cGMP in determining how an axon responds to external stimuli (Song et al., 1998; Song and Poo, 1999). For example, the repulsive response of an axon to the chemorepellent semaphorin 3A (Sema3A) can be switched to an attractive response by increasing the neuron's cGMP levels (Song et al., 1998) and pharmacological inhibition of PKG activity affects Sema3A-induced retinal growth cone responses in *Xenopus laevis* (Campbell et al., 2001). Gyc76C is required for axon guidance in the *Drosophila* embryo, specifically for semaphorin-1a (Sema-1a)-plexin A repulsive guidance of motor axons (Ayoob et al., 2004); however, it is not known how signaling events downstream of Gyc76C directs the axonal response. Based on our studies, Gyc76C may regulate axon guidance by controlling integrin-mediated adhesion and/or interactions with the ECM. In support of this, semaphorin-dependent control of cell migration is known to involve integrin-based adhesion (Pasterkamp and Kolodkin, 2003; Tamagnone and Comoglio, 2004; Tran et al., 2007; Zhou et al., 2008).

## Materials and Methods

### *Drosophila* strains and genetics

Canton-S flies were used as wild-type controls. *lanB2^MB04039^* (referred to here as *LanB2*) was obtained from the Bloomington Stock Center and described in FlyBase (http://flybase.org). *gyc76C^2388^* was generated by standard EMS mutagenesis as previously described (Myat et al., 2005). *DG1^f05504^* was obtained from the Exelixis collection at Harvard Medical School and is described in FlyBase. *gyc76C^Ex173^* and UAS-*gyc76C^WT^* lines were obtained from A. Kolodkin (Johns Hopkins University School of Medicine, Baltimore, MD, USA). The *DG1* and *DG2* RNAi lines were obtained from Shireen Davies (University of Glasgow, United Kingdom) and is previously described (Overend et al., 2012; Vermehren-Schmaedick et al., 2010). *LanA^9–32^* allele was obtained from J. Roger Jacobs (McMaster University, Canada).

### Antibody staining of embryos

Embryo fixation and antibody staining were performed as previously described (Xu et al., 2011). The following antisera were used at the indicated dilutions: mouse βPS and mouse αPS2 antisera (Developmental Studies Hybridoma Bank, DSHB; Iowa City, IA) at 1:200 and 1:5, respectively; rabbit talin antiserum (a kind gift from N. Brown) at 1:500; rabbit laminin α1,2 antiserum (a kind gift from S. Baumgartner) at 1:1000; mouse Crumbs antiserum (DSHB) at 1;20; rat dCREB antiserum at 1:10,000 for DAB staining and 1:2500 for fluorescence staining; and mouse β-galactosidase (β-gal) antiserum (Promega, Madison, WI) at 1:10,000 for DAB staining and 1:500 for fluorescence staining. Appropriate biotinylated- (Jackson Immunoresearch Laboratories, Westgrove, PA), AlexaFluor 488-, 647- or Rhodamine- (Molecular Probes, Eugene, OR) conjugated secondary antibodies were used at a dilution of 1:500. Whole-mount DAB stained embryos were mounted in methyl salicylate (Sigma, St. Louis, MO) and embryos were visualized on a Zeiss Axioplan 2 microscope with Axiovision Rel 4.2 software (Carl Zeiss, Thornwood, NY). Whole-mount immunofluorescence stained embryos were mounted in Aqua Polymount (Polysciences, Inc., Warrington, PA) and thick (1 µm) fluorescence images were acquired on a Zeiss Axioplan microscope (Carl Zeiss) equipped with LSM 510 for laser scanning confocal microscopy at the Weill Cornell Medical College optical core facility (New York, NY).
